# Supplementary material for: Automated vitrification of cryo-EM samples with controllable sample thickness using suction and real-time optical inspection
Source: Nat Commun. 2022 May 27;13:2985. doi: 10.1038/s41467-022-30562-7 (PMC9142589; doi:10.1038/s41467-022-30562-7)
Supplement: Supplementary file 1 — Supplementary Information [file 41467_2022_30562_MOESM1_ESM.pdf]

SUPPLEMENTARY INFORMATION TO:

Automated vitrification of cryo-EM samples with controllable sample thickness using suction and real-time optical inspection.

Roman I. Koning[1*], Hildo Vader[2], Martijn van Nugteren[2], Peter A. Grocutt[2], Wen Yang[3], Ludovic L.R. Renault[3], Abraham J. Koster[1], Arnold C.F. Kamp[2], Michael Schwertner[2]

[1] Electron Microscopy, Cell and Chemical Biology, Leiden University Medical Center, P.O.Box 9600, 2300 RC Leiden The Netherlands.

[2] Linkam Scientific Instruments Ltd, Tadworth, Surrey, KT20 5LR, United Kingdom.

[3] NeCEN, Institute of Biology Leiden, Leiden University, Gorlaeus Building, Einsteinweg 55, 2333 CC Leiden, The Netherlands.

***Corresponding author:** r.i.koning@lumc.nl

Supplementary Figure 1 Single Particle Analysis results

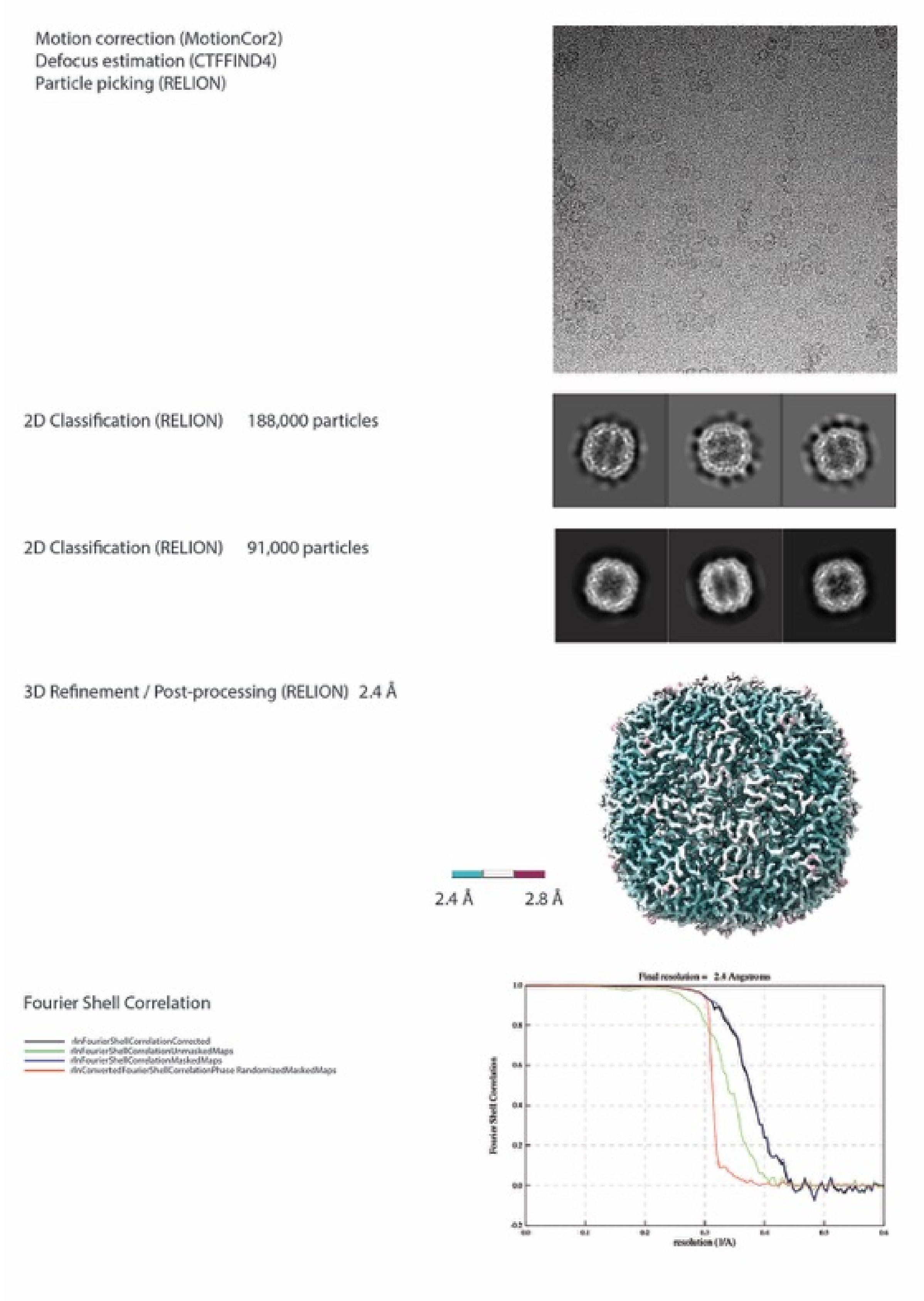

Supplementary Figure 2. Correlation, distribution and quality assessment of sample layer thickness by cryo-EM.

Overviews of seven different grids (columns) showing that the vitrified water layer thickness (as observed by cryo-EM) in the grid squares range from completely dry on the outside of the grid to almost completely filled in the centre of the grid. Top row shows image overlay of last LM movie frame before vitrification on top of a cryo-EM whole grid (multi-image composite) overview of the same grid. The overlay shows that the used 10x lens in LM mode covers most of the grid area that is accessible for TEM imaging. Bottom row shows the LM-covered area of the grid (red rectangles) and grid squares suitable for data collection (good vitrified water thickness and hole covering) marked red. Quantification shows that 30% to 60% of the whole grid and the majority of the area that is visible in LM ends up having a suitable thickness for cryo-EM.

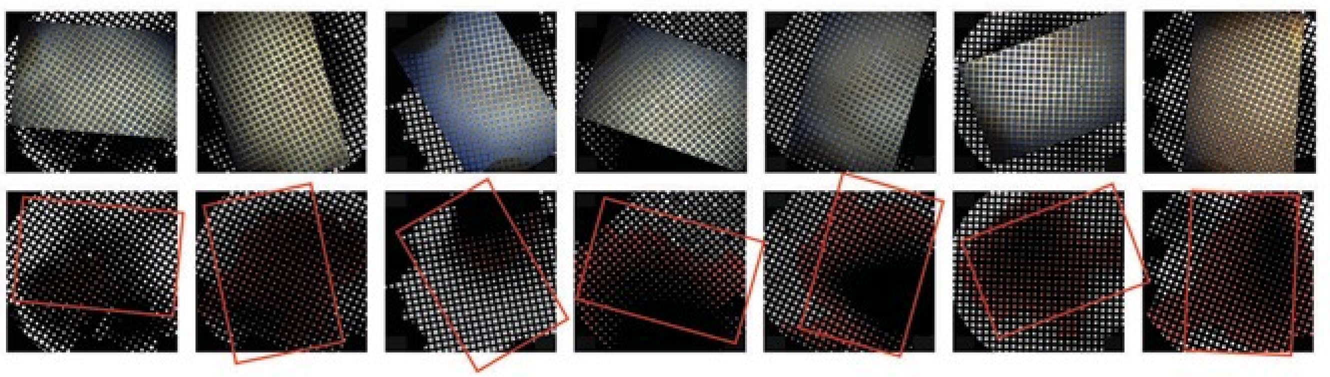

Supplementary Figure 3 Light microscopy observation of protein aggregation

A) Clear spots or B) an overall grainy appearance are typical for presence of larger or smaller aggregates. Also C) Hydration state of cells and D) hydrophilic parts of grids can be observed. E) Two consecutive image stills from movie during water removal show opening up of single 2 micron holes by change of contrast.

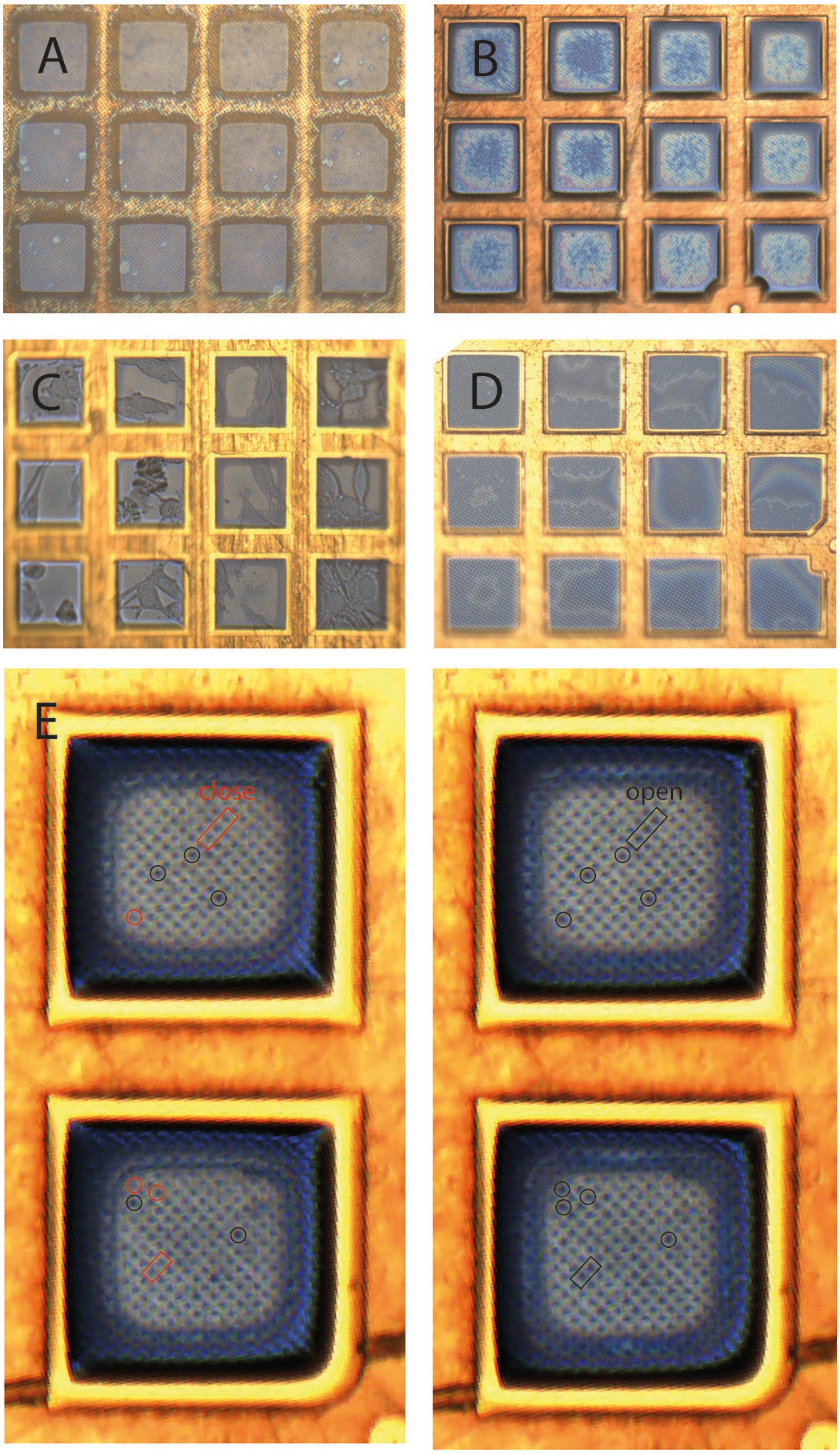

Supplementary Figure 4 Dewpoint

Stills from Supplementary Movie 2 showing stability and control of water layer on surface of grid over the course of 6 minutes. Inset shows single grid square.

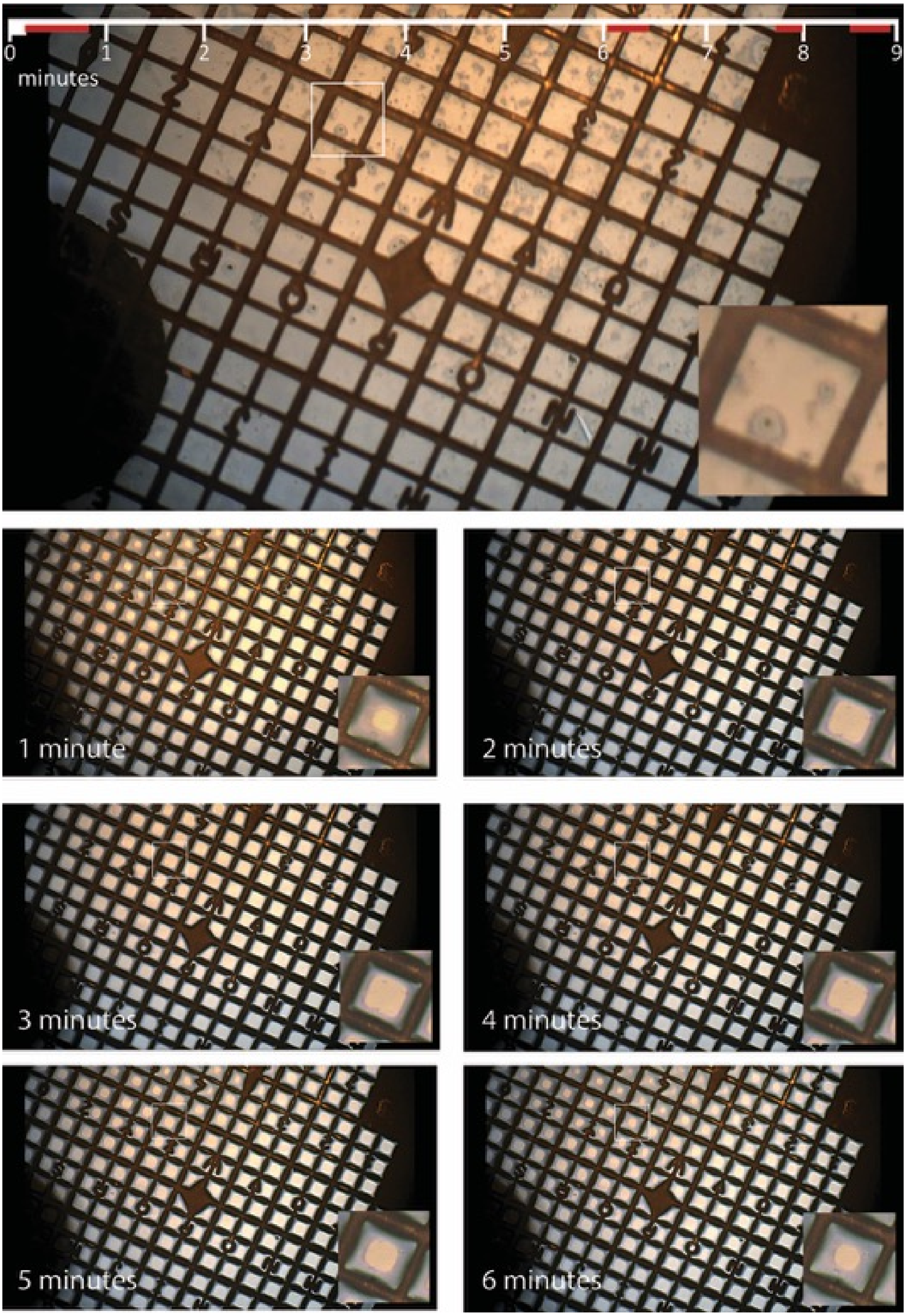

Supplementary Note 1: Protocol

Preparation:

1. Fill water pump with water and connect to plunger.
2. Connect lens heater and connect Peltier (32℃) to T96 controller.
3. Connect Peltier of tweezer lock to power supply and set to 0.20 V and 0.26 A. (Temperature will be about 8 ℃ lower than the incubator chamber).
4. Connect USB cable to laptop and start laptop.
5. Connect main cable and put plunger on.
6. On the laptop open Spinview (camera software).
7. On the laptop open plunger software and connect with plunger.
8. Connect the ethane to the flowmeter.
9. Fill the incubator chamber with a small layer of demineralized water.
10. Fill liquid nitrogen container with 200 mL liquid nitrogen.
11. Press ′′Start LN2′′ and wait until cryogenic chamber is filled.
12. Press ′′Start Ethane′′ and wait until ethane reached -180 ℃.
13. Press ′′Start Autofill′′ and wait until ethane is liquified and the cup is filled.
14. Press ′′Start Incubator′′ and wait until the incubator chamber reached the desired temperature.

Running:

1. Put three grids in the grid box.
2. Put the grid box in the incubator chamber.
3. Fill the de sample container with sample (about 12 uL).
4. Select (software) which grid to pick up (1,2 or 3).
5. Press ′′Load Glow′′. (Skip this step when working with cells grown on grid)
    a. Grid is picked up and transported to glow discharger.
    b. Grid is glow discharged (1.8 mbar, 500 V, 2 mA, 15sec).
6. Press ′′Dip-suction′′
    a. Grid is picked up from glow discharger and transported into the sample container.
    b. Grid is submerged (5 sec) into the sample.
    c. Grid is transported to suction/camera position.
7. Set ′′lightning′′ (transmitted and reflective light) on grid.
8. Focus lens on grid.
9. Position suction tubes onto grid.
10. Press ′′Start-suction′′.
11. Visually follow thickness of water layer.
12. Press ′′Plunge′′.
    a. Grid is plunged into the ′′liquid ethane container′′ and stays there for 5 sec.
    b. Grid is transported to the ′′cryogenic grid storage box′′.
    c. Grid is positioned in first position of "cryogenic grid storage box".
    d. Tweezers are positioned to the ′′Cleaning bath′′ and stays there for 2 sec.
    e. Tweezers go to ′′home′′ position

Turning of:

1. Press ′′stop autofill′′.
2. Press ′′stop LN2′′.
3. Press ′′stop ethane′′.
4. Remove demineralized water from "incubator chamber".
5. Remove and clean media containers.

Supplementary Table 1. Single Particle Data Collection and Processing Parameters

**Parameter**	**SPA**
Magnification	105,000
Pixel size (Å)	0.834
Voltage (kV)	300
Electron exposure (e^-/Å^2)	65
Frames per image	50
Defocus Range (µm)	-0.6 to -2.5
Number of movies collected	3,348
Particles extracted / Final	689,633 / 91,000
Symmetry	O
Masked resolution at FSC=0.143 (Å)	2.42
Map sharpening B-factor (Å^2)	-65

Supplementary Table 2. Comparison of commercial plunge freezing devices

Deposition technique	Device	Methodology	Sample carrier compatibility	Stock volume	Volume per grid	Dewpoint control	Layer inspection	Time from deposition to vitrification	Grid coverage
Grid dipping	Linkam	Liquid film removal via suction and thickness control via flow rate, time and dewpoint control	All	10 µl	<1 µl	Yes	Yes	30-60 s	100%

Characteristics of here described vitrification device. For comparable deposition techniques see Weissenberger et al. [29].
