# Supplementary material for: Automated vitrification of cryo-EM samples with controllable sample thickness using suction and real-time optical inspection
Source: Nat Commun. 2022 May 27;13:2985. doi: 10.1038/s41467-022-30562-7 (PMC9142589; doi:10.1038/s41467-022-30562-7)
Supplement: Supplementary file 3 — Description of Additional Supplementary Files [file 41467_2022_30562_MOESM3_ESM.docx]

**Description of Additional Supplementary Files**

**File Name: Supplementary Data 1**

**Description:** ssDNA sequences for DNA origami.

**File Name: Supplementary Movie 1
Description:** Schematic sequence of automated events showing for the Linkam plunger.

**File Name: Supplementary Movie 2
Description:** Light microscopy movie of controlling water layer thickness using dewpoint control.

**File Name: Supplementary Movie 3
Description:** Typical light microscopy view of grid during water layer thinning by suction.
