# Supplementary material for: Automated vitrification of cryo-EM samples with controllable sample thickness using suction and real-time optical inspection
Source: Nat Commun. 2022 May 27;13:2985. doi: 10.1038/s41467-022-30562-7 (PMC9142589; doi:10.1038/s41467-022-30562-7)
Supplement: Supplementary file 8 — Reporting Summary [file 41467_2022_30562_MOESM8_ESM.pdf]

nature portfolio

Corresponding author(s): R.I.Koning

Last updated by author(s): Apr 22, 2022

Reporting Summary

Nature Portfolio wishes to improve the reproducibility of the work that we publish. This form provides structure for consistency and transparency in reporting. For further information on Nature Portfolio policies, see our Editorial Policies and the Editorial Policy Checklist.

Statistics

For all statistical analyses, confirm that the following items are present in the figure legend, table legend, main text, or Methods section.

n/a	Confirmed	
☐	☒	The exact sample size (*n*) for each experimental group/condition, given as a discrete number and unit of measurement
☒	☐	A statement on whether measurements were taken from distinct samples or whether the same sample was measured repeatedly
☒	☐	The statistical test(s) used AND whether they are one- or two-sided<br>*Only common tests should be described solely by name; describe more complex techniques in the Methods section.*
☒	☐	A description of all covariates tested
☒	☐	A description of any assumptions or corrections, such as tests of normality and adjustment for multiple comparisons
☒	☐	A full description of the statistical parameters including central tendency (e.g. means) or other basic estimates (e.g. regression coefficient) AND variation (e.g. standard deviation) or associated estimates of uncertainty (e.g. confidence intervals)
☒	☐	For null hypothesis testing, the test statistic (e.g. *F*, *t*, *r*) with confidence intervals, effect sizes, degrees of freedom and *P* value noted<br>*Give P values as exact values whenever suitable.*
☒	☐	For Bayesian analysis, information on the choice of priors and Markov chain Monte Carlo settings
☒	☐	For hierarchical and complex designs, identification of the appropriate level for tests and full reporting of outcomes
☒	☐	Estimates of effect sizes (e.g. Cohen's *d*, Pearson's *r*), indicating how they were calculated

Our web collection on statistics for biologists contains articles on many of the points above.

Software and code

Policy information about availability of computer code

Data collection	Thermo Fischer Scientific: EPU v.4; Tomography v.4; MAPS v.3; Teledyne Spinnaker/Spinview v 1.1.10.43
Data analysis	Amira 2020.2 - Gatan: GMS v.3 - UCSF Chimera v 1.13 and X v 1.0 - Relion: Gaussian Picker; 3.1 (beta); Motioncor2 - CTFFIND 4.1.18 - IMOD 4.11 - Zeiss Zen Blue 3.4 - Adobe: Photoshop 2021; Premiere Pro 2020; Maxon Cinema 4D R22; Maxon Redshift v 3

For manuscripts utilizing custom algorithms or software that are central to the research but not yet described in published literature, software must be made available to editors and reviewers. We strongly encourage code deposition in a community repository (e.g. GitHub). See the Nature Portfolio guidelines for submitting code & software for further information.

Data

Policy information about availability of data

All manuscripts must include a data availability statement. This statement should provide the following information, where applicable:

- Accession codes, unique identifiers, or web links for publicly available datasets
- A description of any restrictions on data availability
- For clinical datasets or third party data, please ensure that the statement adheres to our policy

The apoferritin cryo-EM map generated in this study have been deposited in the EMDB under accession code EMD-13738 (https://www.emdataresource.org/EMD-13738). The data that support this study are available from the corresponding authors upon reasonable request.

Field-specific reporting

Please select the one below that is the best fit for your research. If you are not sure, read the appropriate sections before making your selection.

☒ Life sciences ☐ Behavioural & social sciences ☐ Ecological, evolutionary & environmental sciences

For a reference copy of the document with all sections, see nature.com/documents/nr-reporting-summary-flat.pdf

Life sciences study design

All studies must disclose on these points even when the disclosure is negative.

Sample size	No sample size was calculated. For each sample per experiment the sample size is set by the specimen preparation protocol. For single particle analysis and and electron tomography on particle suspensions, 3 microliter of purified sample is added to the grid and vitrified. For cellular electron microscopy cells are grown on an EM grid surface and suitable cells are chosen from that.
Data exclusions	No data was excluded from the studies.
Replication	In total 18 separate experiments were performed to test functionality and acquire data and replicate results took place during the development of the device. After finalizing the functionality of the device, replication of data after this was done once or twice, all with positive and similar results.
Randomization	We did not do randomization. We did test as many different types of samples to rule out sample bias in the use and functionality of the device. E.g, bacteria are easy to vitrify while liposomes / vesicles can be very sensitive to circumstances.
Blinding	Not relevant. We did not perform statistical or clinical trials. Blinding experiments during the development of the plunger is not desired since we needed the outcome for improving the performance. When we tested the functional version of the plunger we did not use blinding of results compared to existing apparatuses, since we did not perform comparative studies. Furthermore we performed no blinding in the sense that we had multiple persons using the apparatus, since hardware and software was still in development phase.

Reporting for specific materials, systems and methods

We require information from authors about some types of materials, experimental systems and methods used in many studies. Here, indicate whether each material, system or method listed is relevant to your study. If you are not sure if a list item applies to your research, read the appropriate section before selecting a response.

Materials & experimental systems

n/a	Involved in the study	
☐	☒	Antibodies
☐	☒	Eukaryotic cell lines
☒	☐	Palaeontology and archaeology
☒	☐	Animals and other organisms
☒	☐	Human research participants
☒	☐	Clinical data
☒	☐	Dual use research of concern

Methods

n/a	Involved in the study	
☒	☐	ChIP-seq
☒	☐	Flow cytometry
☒	☐	MRI-based neuroimaging

Antibodies

Antibodies used	IgG1 DNP monoclonal antibodies (gift from collaborators, preparation as described in the reference below)
Validation	https://journals.plos.org/plosbiology/article?id=10.1371/journal.pbio.1002344

Eukaryotic cell lines

Policy information about cell lines

Cell line source(s)	17Clone1 mouse cells (https://journals.plos.org/plosbiology/article?id=10.1371/journal.pbio.3000715)
Authentication	Not authenticated
Mycoplasma contamination	Cell line was tested negative for mycoplasma
Commonly misidentified lines (See ICLAC register)	no
